# Code of practice for medical autopsies: a minimum standard position paper for pathology departments performing medical (hospital) autopsies in adults

**DOI:** 10.1007/s00428-021-03242-y

**Published:** 2021-12-10

**Authors:** G. Cecilie Alfsen, Jacek Gulczyński, Ivana Kholová, Bart Latten, Javier Martinez, Myriam Metzger, Katarzyna Michaud, Carlos M. Pontinha, Natalia Rakislova, Samuel Rotman, Zsuzsanna Varga, Katharina Wassilew, Vsevolod Zinserling, on behalf of Working group Autopsy Pathology, European Society of Pathology

**Affiliations:** 1 Department of Pathology, Akershus University Hospital, Loerenskog, Norway; 2 Faculty of Medicine, University of Oslo, Oslo, Norway; 3 Department of Pathology and Neuropathology, Medical University of Gdańsk, Gdansk, Poland; 4 Pathology, Fimlab Laboratories, Faculty of Medicine and Health Technology, Tampere University, Tampere, Finland; 5 Department of Forensic Pathology, Netherlands Forensic Institute, The Hague, The Netherlands; 6 Department of Pathology, Maastricht University Medical Center+, Maastricht, The Netherlands; 7 Servicio de Anatomía Patológica, Complejo Asistencial Universitario de León (CAULE), Leon, Spain; 8 Department of Pathology, Saarland University Medical Center, Homburg/Saar, Germany; 9 University Center of Legal Medicine Lausanne - Geneva, Lausanne, Switzerland; 10 Lausanne University Hospital and University of Lausanne, Lausanne, Switzerland; 11 Department of Anatomical Pathology, Central Lisbon University Hospital Centre, Lisbon, Portugal; 12 ISGlobal, Barcelona Institute for Global Health, Hospital Clínic-Universitat de Barcelona, 08036 Barcelona, Spain; 13 Service of Clinical Pathology, Lausanne University Hospital, University of Lausanne, Lausanne, Switzerland; 14 Department of Pathology and Molecular Pathology, University Hospital Zurich, Zurich, Switzerland; 15 Department of Pathology, Rigshospitalet, Copenhagen, Denmark; 16 Department of Pathomorphology, Institute of Experimental Medicine V.A. Almazov National Research Center, Saint Petersburg, Russian Federation

**Keywords:** Autopsy, Guideline, Quality, Turnaround time, Standards

## Abstract

The medical autopsy (also called hospital or clinical autopsy) is a highly specialised medical procedure, which requires professional expertise and suitably equipped facilities. To ensure high standards of performance, the Working Group of Autopsy Pathology of the European Society of Pathology (ESP) suggests a code of practice as a minimum standard for centres performing medical autopsies. The proposed standards exclusively address autopsies in adults, and not forensic autopsies, perinatal/or paediatric examinations. Minimum standards for organisation, standard of premises, and staffing conditions, as well as minimum requirements for level of expertise of the postmortem performing specialists, documentation, and turnaround times of the medical procedure, are presented. Medical autopsies should be performed by specialists in pathology, or by trainees under the supervision of such specialists. To maintain the required level of expertise, autopsies should be performed regularly and in a number that ensures the maintenance of good practice of all participating physicians. A minimum number of autopsies per dedicated pathologist in a centre should be at least 50, or as an average, at least one autopsy per working week. Forensic autopsies, but not paediatric/perinatal autopsies may be included in this number. Turnaround time for final reports should not exceed 3 weeks (14 working days) for autopsies without fixation of brain/spinal cord or other time-consuming additional examinations, and 6 weeks (30 working days) for those with fixation of brain/spinal cord or additional examinations.

## Introduction

The medical autopsy is a postmortem external and internal examination of the human body, performed with the intention of identifying or confirming cause of death and underlying diseases. In addition, the medical autopsy is of major importance for bereavement follow-up, quality assurance of clinico-medical practice, monitoring of diseases and treatments, cause of death statistics and public health monitoring, rehearsal of medical procedures, education of medical students and other types of health personnel, and for research purposes. The recent pandemic has confirmed the important role of autopsies in the understanding of diseases (Table 1).

**Table 1:** The medical autopsy: contributions to evidence-based medicine

Area	Comment
Understanding of diseases	Historically, autopsies form the basis of medical knowledge. Less acknowledged is the fact that autopsies remain essential for the follow-up of diseases, and for uncovering both common [52] and rare diseases, among others within the field of neuropathology (f.e. prion diseases [53], Alzheimer’s [54], multiple sclerosis [55]), infectious diseases (as Legionnaire’s disease [56], AIDS [57, 58], SARS [59], COVID-19 [60, 61]), collagen vascular disorders [62], to name a few
Quality control of clinical practice	Despite advances in laboratory medicine and diagnostic imaging, autopsies continue to reveal unsuspected, clinically important diagnoses in a significant number of deaths [63, 64]. Low autopsy rates may lead to an overestimation of clinical diagnostic performance [65]
Teaching of medical students, residents, physicians, and other healthcare staff	Besides pathology, pathological anatomy, topography, and pathophysiology, also clinicopathological correlations and observations are core skills learnt from autopsies [66]. The declining autopsy numbers raise concerns about future medical training, and the quality of diagnosis and medical treatment [67, 68]
Training in medical procedures	Training on corpses is an invaluable tool for rehearsal of complex anatomy for surgical procedures [69, 70]. The legal regulation of post-mortem clinical training practice is thus often included in autopsy legislations [71]
Quality control of public statistics	Being considered “gold standard” for cause of death, autopsy results are used for correction of public statistics [72]. Low autopsy rates worldwide endanger adequate distribution of funding of various diseases [73, 74]
Tissue sampling and research	Autopsies remain a major source for sampling of fluids, cells, and tissues. As a substitute for the full, conventional post-mortem, f.e. in cases of consensual problems, or in need for ultra-fresh sampling, minimally invasive procedures, and rapid research autopsy have been developed [75, 76]

The medical autopsy is a highly specialised medical procedure which requires professional expertise and suitably equipped facilities. From a historical view, autopsies have formed the basis of evidence-based medicine [1, 2]. However, while evidence-based medicine has undergone further developments in line with the general development of society and science, the number of autopsies has declined sharply [3, 4]. The explanations for the decline of autopsy practice are manifold and include among others, differences in legislation, rejection of autopsy requests due to religious motivation, traditions, or simply insufficient funding on provision of postmortem examination by public health systems in some countries [5, 6]. Of major importance is also the lack of focus on postmortem examinations from site of the clinicians, who, on the background of increasing work-load pressure, lack both time and knowledge for autopsy-related communications with next of kin, have a perception of reduced value of postmortem examinations due to advancements in medicine, and are discouraged by long turnaround times [3, 7, 8]. Lastly, there is the lack of interest in performing postmortem examinations from the pathologists themselves, who are overburdened by the daily diagnostic routine [3, 9].

A revival of the medical autopsy will require adjustments to and efforts on all the abovementioned factors, but will also have to focus on high standards of performance and quality of the procedure itself. Thus, as a first step in the revival process, common guidelines with the aim of standardizing the procedure are essential.

### Current situation

With the exception of more or less updated guidelines in textbooks on autopsy pathology, published minimum standards for the medical autopsy procedure are few and mostly presented as detailed instructions on how to write the reports, or as scenario-based and forensically related guidelines [10, 11]. Existing guidelines for the investigation of single organs, as the heart or central nervous system, are important for the individual examination, but also do not elaborate on requirements on an overall level, such as organization, premises, or requirements for professional expertise [12, 13, 14]. National or institutional guidelines are predominately published as internet resources and are not only burdened by the linguistic diversities in Europe but also by local adaptions due to different traditions and variable autopsy practices [15, 16, 17].

The widely varying practice of autopsy performance in Europe raised concerns in the working group Autopsy pathology (WGA) of The European Society of Pathology (ESP). A survey among the ESP members showed general shortcomings in the knowledge on local or national autopsy rates, a fact proven by serious shortcomings in European statistics, which fail to depict autopsy activity in major European member States [18, 19]. The large societal differences within Europe make it difficult to unify certain autopsy procedures, as for instance, requisitioning routines. Attempts of standardization of layout of reports have been difficult to pursue also at a national level [20]. However, it is feasible to outline a set of minimum standards with regard to the actual execution of the procedure. This may represent the beginning of the process of ensuring quality, with the aim of regaining the trust of clinicians in the medical autopsy procedure.

### Recommendations, not injunctions

With the purpose of presenting a set of minimum standards and recommendations for the performance of medical autopsies within Europe, WGA appointed in 2019 an ad hoc working group with expert members from ten European countries, working in academic and non-academic institutions. The adopted standards of the WGA are outlined below, after having been subject to discussion at the 32nd Congress of the ESP and XXXIII International Congress of the IAP [21].

It is important to point out that these guidelines reflect current best practice and are subject to constant evaluation. Due to limited published research on the importance of quality of medical autopsies in recent years, it is not possible to grade any of the recommendations. Therefore, they are based on the professional experiences of the members of WGA.

It is emphasized that the proposed standards for adult autopsy service intend to support pathologists in the quest for more resources for this important part of medical quality assurance and shall not be understood as restrictions to perform autopsies if proposed minimum standards are not met by smaller departments.

The suggested minimum standards are primarily developed for autopsies in adults, and not for forensic autopsies or perinatal/paediatric examinations, although similarities are obvious.

### Minimum standards, proposal

#### Organisation

A first step in the revival process is to raise awareness for medical autopsies to colleagues within the own institution, and address the public. Hospital administrators recognizing the potential of autopsies in the hospital profiling on medical quality are as essential as dedicated pathologists, maintaining close collaboration with the clinicians and promoting knowledge in the wider public. The abovementioned approach has proven effective. A Swiss study has shown that raising awareness for the benefits of postmortem examination in the clinical departments by involving their management, combined with a systematic teaching of interns and residents about the importance of autopsy, resulted in doubling of autopsy rates within 6 months [7]. Building a dedicated postmortal diagnostic team, with a limited number of pathologists, and thus optimizing the pathologist-clinician relationship, was part of another Swiss study on reorganizing the autopsy practice [22].

Historically, the medical autopsy procedure has been performed or supervised by any specialist in pathology, to whom competence has been assigned automatically after performance of a defined minimum number of autopsies during their pathology training. In consequence of the rapid decline in autopsy rates, the curriculum of core competency training requirements in pathology has been adjusted to a lower number of postmortem examinations. Contrary to the recommendation for basic competence in autopsy practices outlined by the European Union of Medical Specialists (UEMS), several European countries have removed the minimum requirements for autopsy training in its entirety [18, 23]. Thus, one can no longer assume that specialists in pathology have basic expertise in autopsy practices. Lack of basic knowledge on autopsy work is one of the reasons for advocating autopsy pathology as a subspecialty, a measure that has been implemented in the UK [24, 25, 26].

Quality is a product of high throughput training, and quantity is a result of organization and visibility. The classical medical autopsy is performed in hospitals all over Europe, and with great variation in frequency. UEMS recommends an autopsy frequency as low as 5–10% of hospital deaths, an estimate based on the continuously declining numbers [23]. There are no evidence-based numbers defining the optimal frequency for maintaining a sufficient level of competency within the wide field of postmortem procedures. Our suggestion for a minimum level of activity reflects the view that the procedure should be performed by a dedicated and restricted group of specialists in pathology, who perform autopsy work as part of their daily routines.

- Centres performing medical autopsies should be part of a medical institution and have a dedicated staff, administration, and facilities.
- The autopsy practice should be identifiable in the organizational chart of the institution, and the institutional role of the autopsy procedure stated in policy documents.
- Yearly reports of the autopsy activity should be published on the institutional website and should include a number of autopsies (in total and per performing pathologist/resident in pathology), autopsy rates, and turnaround times and should include any research activities.
- Information material suitable for the public should be easily available on the institutional website.
- The autopsy practice should be under the leadership of a specialist in pathology with sufficient experience in autopsy practice.
- The medical autopsy should be integral part of clinico-pathologic conferences.
- Medical autopsies should only be performed by a specialist in pathology, or by a trainee supervised by a specialist in pathology.
- To maintain expertise, autopsies should be performed regularly and in a number that ensures the maintenance of good practice of all participating physicians. A minimum number of autopsies per pathologist in the autopsy-performing group in a centre should be at least 50, or as an average, at least one autopsy per working week. Forensic autopsies, but not perinatal autopsies may be included in this number.

#### Staffing

The ongoing SARS-CoV-2 pandemic has demonstrated the importance of sufficient, competent, and dedicated staffing, at all times and levels [27]. Autopsy work requires not only personnel that is able to work in teams, and who are considerate of and able to relate to the potential risk of injuries and infections. Requirements in particular for autopsy technicians also include knowledge of pathological anatomy, training in a manifold of dissection techniques, as well as physical and mental strength, to mention some. According to non-published data from the WGA survey on autopsy practice in Europe, less than half of the respondents reported formalized and publicly approved educations for autopsy technicians in their countries [18]. Increasing the formal competence of the aforementioned professional group is therefore of utmost importance.

Not only autopsy staff members, but also other visitors as clinicians and medical students, are exposed to potential infections in the autopsy room. To minimize the risk of inflicted disease, recommended basic vaccination programs should be followed by all autopsy-attending individuals. Staff members with chronically reduced immune systems should not attend regular work in the autopsy room, unless medically approved.

Vaccination recommendations for medical professionals are constantly subject to reevaluation. The recommended vaccinations in public health programs also vary between the different European regions [28]. However, the fact that medical personnel, in relation to the general public, is more exposed to infections and may spread disease to vulnerable patients, highlights the need for a strict follow-up of the vaccination programs in this group.

- Centres performing medical autopsies should have designated permanent staff, of a size ensuring the level of activity.
- Job descriptions should be available for all personnel groups and should include required education, norms for ethical standards, rules for conflict of interests, and confidentiality.
- All personnel in contact with the deceased or with access to autopsy facilities must follow recommended occupational health surveillance programs (vaccination, infectious disease serology, tuberculosis surveillance).
- Procedures in the event of accidental contamination should be outlined and easily available.

#### Premises

The autopsy procedure should be recognized as a form of medical examination, which, although not performed during the patient’s lifetime, requires basic health facility precautions. A revival of the autopsy thus should be reflected in a modernization of facilities, especially in regard to hygiene and safety, storage possibilities, and the implementation of adequate IT tools. The SARS-CoV-2 pandemic has highlighted the need for an update on personal protection and hygiene measures in the autopsy room [29, 30, 31, 32]. The universal precautions as outlined by the Royal College of Pathologists should be followed in all types of autopsies.

- Centres performing medical autopsies should have premises dedicated to and suitable for autopsy procedures at a minimum of biosafety level 2, with controlled access to the autopsy rooms. The centres should have the possibility to reject autopsies, for instance, when biological risk is found too high for protection of their personnel or equipment of facilities. In case of rejection of a request for autopsy, the centre should provide alternative approaches (e.g., minimally invasive autopsy) or refer the autopsy request to another centre with sufficient biosafety level.
- The transportation of bodies from and to the morgue should be performed in areas not accessible to the public.
- Wardrobes with shower facilities must be available.
- Eyewash and other necessary first aid equipment must be present in the autopsy room.
- Security rules and procedures in case of accidents must be easily accessible.
- Protective clothing (surgical scrub suit, waterproof gowns, and plastic aprons), clear visor, gloves (disposable with long sleeves and protective), boots, and FFP3 masks should be used at all times.
- Tools and equipment should be easy to clean and provided with mechanical protection and suction devices, to avoid unnecessary exposition to aerosols, dust, and injuries. Disposable equipment should be available.
- The facilities should have systems for storage of fixed and frozen biomaterials, and easy access to freezers (− 20 °C and − 80 °C).
- Fixation fluids (formalin) and fixated materials should be handled in ventilated areas.
- Cameras and safe IT storage facilities for photographic documentation should be provided.
- Autopsy facilities geographically separate from the procedure-requesting clinicians should be adequately equipped for a live video demonstration of gross findings and postmortem meetings.

#### Additional expertise

Ancillary testing may be crucial in achieving a correct postmortem diagnosis. A Norwegian study on the quality of medical autopsies in 2014 found that ancillary testing for viral infections was rare [9]. One explanation for the lack of application of testing or expertise from other specialties may be additional costs [33]. Another reason is the risk of delaying the final report. The examination of the brain by a neuropathologist is a known cause for prolonged turnaround times and reflects limited capacity of neuropathology expertise [9]. To avoid unnecessary strain on scarce resources, ancillary testing or involvement of additional expertise should be performed according to defined criteria.

- Centres performing medical autopsies should have access to necessary additional analytic methods and/or expertise from other specialties, especially within microbiology, toxicology, clinical chemistry, and radiology.
- Access to expertise from specialised areas within pathology, in particular within neuropathology, cardiovascular pathology, and forensic pathology, should be ensured.
- Guidance in the use of ancillary testing or involvement of additional expertise should be incorporated in guidelines.

#### Other guidelines

Written autopsy guidelines should be an integral part of department guidelines. Up-to-date guidelines are important at a number of levels and help prevent errors and waste of resources.

Centres performing medical autopsies should have written guidelines on:

- hygiene and biosafety procedures, including disposal of material, bodily fluids, and tissues
- safe storage of requisitions, reports, results, biomaterials
- morgue procedures
- communication with next of kin, clinicians, and non-medical personnel (undertakers, police etc.)
- procedures in questions of hereditary diseases and genetic examinations
- different types of autopsy scenarios, including possible forensic scenarios (for instance sudden death, postoperative deaths, unexpected findings during autopsy requiring forensic expertise)

#### Demonstrations and reporting

The involvement of clinicians in the immediate aftermath of the procedure is important for the discussion of findings and ensures that one can still return to the body, should new information evolve. The clinico-pathological correlation and open discussion of discrepancies therein represent a valuable tool for improving communication with the clinicians, in addition to the educational aspect [34, 35, 36, 37, 38].

A standardization of the reporting ensures provision of all important elements and eases the reading and comparison of clinico-pathological findings, also in regard to future research [10,20, 39, 40, 41]. There is a universal agreement on the need for following the World Health Organization (WHO) guidelines when stating the cause of death [42, 43]. However, there are differences in the view of whether only patho-anatomical diagnoses should be included, or if pathologists in addition should consider clinical findings. The College of American Pathologists (CAP) advises against the random listing of anatomic diagnoses and encourages the inclusion of clinical information when construction the cause of death [10]. The German group of Wittekind and colleagues suggests to cite the clinical cause of death and patho-anatomical cause of death separately, a procedure which eases comparability, not least for the cause of death-registries [20]. A prerequisite for both suggestions is access to the full clinical story of the deceased. As the practice of authoring death certificates varies, with pathologists in eastern European countries with traditionally high autopsy coverage issuing the major part of death certificates, the German recommendation is not practicable in all parts of Europe. Regardless of type of findings stated as the cause of death in the autopsy report, it should be clarified which diagnoses are based on patho-anatomical findings and which are not.

Not all elements of the final report are required to be forwarded to the clinicians. One may consider which parts of the report are necessary to substantiate the conclusion. One example is photographic documentation, in case the autopsy report is being made available to the next of kin of the deceased. However, to allow the clinicians to validate the autopsy procedure and the diagnoses given, the report should as a minimum contain information about all types of sampling, tests, and documentations.

There are examples of national jurisdictional provisions on the consent that may cause problems in obtaining samples for microscopy routinely [44]. Jurisdictions are not the subject of this paper. However, it is emphasized that microscopic examination represents an integral part of a complete medical autopsy. Thus, microscopic descriptions are part of the listed minimum documentations.

- Gross findings should be demonstrated to clinicians immediately after each autopsy procedure, if necessary by means of photographic or multimedia documentation. A preliminary written report should be given within two working days after the autopsy.
- The preliminary report should list all findings in an orderly manner and should address each clinical question. The preliminary report should include an estimate of the expected turnaround time of the final report.
- The final report should be in the form of a standardized autopsy protocol, ensuring a standardized setup of gross organ descriptions and areas and microscopic descriptions of studied organs and tissues.
- Cause of death, including chain of events from underlying disease to immediate cause of death or condition, and other major diagnoses and findings should be reported according to the WHO guidelines/International Statistical Classification of Diseases and related health problems (ICD).
- If necessary, additional comments should be given to explain the chain of events leading to the death of the patient, to answer any questions of the clinician, and/or to comment on major differences between the clinically estimated cause of death and patho-anatomical findings.

##### *Minimum documentation in final report*

- ID of body, date of birth, date of death, consignor (name and address), date and time of autopsy, ID nr of autopsy, name of pathologist, and autopsy technician.
- Legal requirements (written consent, contact with police etc.).
- Purpose of autopsy/clinical question.
- Clinical history.
- Type of autopsy (complete/partial).
- External examination.
- Internal examination, including gross description.
- List of sampled organs and/or lesions.
- Location of tissue samples taken for microscopy and their identification (block ID).
- Microscopic descriptions.
- Ancillary studies (toxicology, bacteriology etc.) and their results .
- Any photographic documentation.
- Any retained organs, with an explanatory note about the reasons for retainment.
- Other types of storage (fluids, tissues, or swabs).
- An addendum should contain a reference to the relevant legislation and give a general description on the storage length and timeline of destruction of biological material and remnants from the autopsy.

### Turnaround time for final report

In recent years, turnaround times of autopsy reporting are generally too long, although being essential for clinicians and the bereaved, and representing important indicators of quality [9, 24, 45, 46, 47]. The sometimes excessive delay in reporting autopsy results has been interpreted as a lack of motivation by the pathologists [48]. Several studies have successfully shortened the turnaround times by reorganising the workflow and space organization [22, 49, 50]. To shorten turnaround times by releasing the report before supplemental reports as for instance neuropathology and microbiology are issued has been suggested by CAP and also applied with success by centres in Europe [10, 51].

Guidelines on turnaround times vary. CAPs guidelines recommend issuing of the final report within 30 working days for routine cases and 90 days for complex cases, although the latter is not defined [10]. The suggested turnaround time from the German group is 4 weeks for all autopsy procedures of various complexity, while the Norwegian Society of Pathology has decided on 2 weeks for regular autopsies without neuropathology examination, and 2 months including neuropathological examination [15, 20].

If a revival of the medical autopsy is to succeed, the procedure must be considered equal to a medical examination. The processing of autopsy material does not differ from tissues from the living but is often not considered a priority. The WGA is the opinion that turnaround times for medical autopsies should be defined according to what is technically possible to achieve, not according to what is practised today. The recommended turnaround times do not include the day of autopsy performance.

- Medical autopsies without fixation of the brain/spinal cord or additional examinations should be reported within a maximum of 3 weeks (14 working days).
- Medical autopsies with fixation of brain/spinal cord and/or additional examinations should be reported within a maximum of 6 weeks (30 working days).
